# The Essential Element Manganese, Oxidative Stress, and Metabolic Diseases: Links and Interactions

**DOI:** 10.1155/2018/7580707

**Published:** 2018-04-05

**Authors:** Longman Li, Xiaobo Yang

**Affiliations:** ^1^Department of Occupational Health and Environmental Health, School of Public Health, Guangxi Medical University, Nanning, Guangxi, China; ^2^Center for Genomic and Personalized Medicine, Guangxi Medical University, Nanning, Guangxi, China

## Abstract

Manganese (Mn) is an essential element that is involved in the synthesis and activation of many enzymes and in the regulation of the metabolism of glucose and lipids in humans. In addition, Mn is one of the required components for Mn superoxide dismutase (MnSOD) that is mainly responsible for scavenging reactive oxygen species (ROS) in mitochondrial oxidative stress. Both Mn deficiency and intoxication are associated with adverse metabolic and neuropsychiatric effects. Over the past few decades, the prevalence of metabolic diseases, including type 2 diabetes mellitus (T2MD), obesity, insulin resistance, atherosclerosis, hyperlipidemia, nonalcoholic fatty liver disease (NAFLD), and hepatic steatosis, has increased dramatically. Previous studies have found that ROS generation, oxidative stress, and inflammation are critical for the pathogenesis of metabolic diseases. In addition, deficiency in dietary Mn as well as excessive Mn exposure could increase ROS generation and result in further oxidative stress. However, the relationship between Mn and metabolic diseases is not clear. In this review, we provide insights into the role Mn plays in the prevention and development of metabolic diseases.

## 1. Introduction

**Figure 1:**
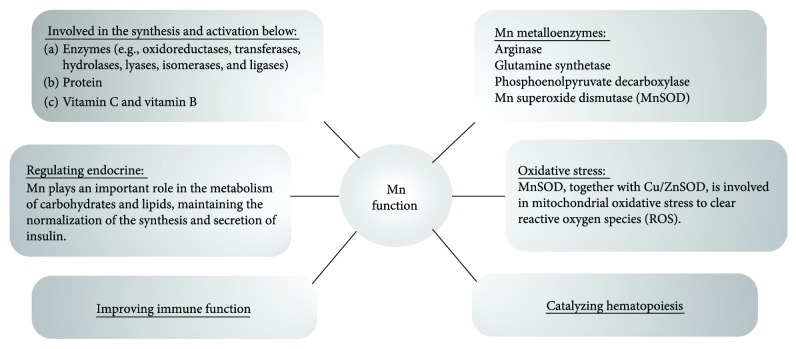
Physiological roles of Mn.

Manganese (Mn) is an essential element in the human body that is mainly obtained from food and water. Mn is absorbed through the gastrointestinal tract and then transported to organs enriched in the mitochondria (in particular the liver, pancreas, and pituitary) where it is rapidly concentrated [1]. Furthermore, Mn is involved in the synthesis and activation of many enzymes (e.g., oxidoreductases, transferases, hydrolases, lyases, isomerases, and ligases); metabolism of glucose and lipids; acceleration in the synthesis of protein, vitamin C, and vitamin B; catalysis of hematopoiesis; regulation of the endocrine; and improvement in immune function [2]. Moreover, Mn metalloenzymes including arginase, glutamine synthetase, phosphoenolpyruvate decarboxylase, and Mn superoxide dismutase (MnSOD) also contribute to the metabolism processes listed above and reduce oxidative stress against free radicals (Figure 1).

However, environmental or occupational Mn overexposure is harmful to human health, especially in at-risk populations such as miners, welders, and steel makers. According to data from the Mineral Commodity Summaries released by the US Geological Survey in 2016, South Africa, China, and Australia accounted for 67% of the total Mn mined (18 million tons) in the world in 2015. Mn ore mining and its processing cause air and water pollution, threatening the health of workers and general populations residing near factories through oral ingestion and inhalation as well as dermally and intravenously. Acute Mn exposure can lead to manganism, and chronic Mn exposure causes an extrapyramidal syndrome with features resembling those found in Parkinson's disease and postencephalitic parkinsonism [3].

The prevalence of metabolic diseases, including type 2 diabetes mellitus (T2DM), obesity, insulin resistance, atherosclerosis, hyperlipidemia, nonalcoholic fatty liver disease (NAFLD), and hepatic steatosis, has increased dramatically over the past few decades [4]. These metabolic disorders are usually caused by the clustering of metabolic syndrome (MetS). The criteria for identifying MetS include three of five markers: abdominal obesity, impaired carbohydrate metabolism, high blood pressure, and dyslipidemia, including elevated levels of triglycerides and decreased levels of high-density lipoprotein (HDL) [5]. In addition, many studies have shown that metabolic diseases are associated with oxidative stress and inflammation [6–12].

Mn is a component or activator of some enzymes, mostly antioxidants, and plays an important role in metabolisms of carbohydrates and lipids, even in maintaining the normalization of the synthesis and secretion of insulin as well. Therefore, Mn may have protective effects on the occurrence of MetS [13].

**Figure 2:**
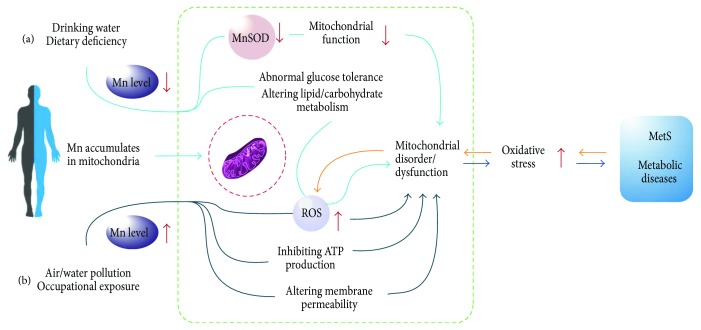
The mechanisms of Mn in metabolic diseases via oxidative stress. (a) Mn deficiency will cause a number of detrimental effects, such as impaired growth, poor bone formation and skeletal defects, reduced fertility and birth defects, abnormal glucose tolerance, and altered lipid and carbohydrate metabolism in both animals and humans. Therefore, Mn deficiency might lead to mitochondrial dysfunction or disorder via decreasing MnSOD level and altering lipid and carbohydrate metabolism. (b) Mn overloaded may disrupt normal mitochondrial function by increasing mitochondrial ROS, inhibiting ATP production, and altering membrane permeability; further result in mitochondrial dysfunction or disorder; and finally cause MetS or metabolic diseases. Excessive ROS and oxidative stress would lead to MetS or metabolic diseases directly. If MetS or metabolic diseases happen, it will in turn increase ROS production and oxidative stress and accelerate mitochondrial dysfunction or disorder.

Importantly, Mn is a required component of MnSOD for reducing mitochondrial oxidative stress. Mitochondria are the major place where physiological and pathological cellular reactive oxygen species (ROS) are produced. When excessive ROS accumulate abnormally, it would contribute to the oxidative damage found in several neuropathological conditions related to enhanced glucocorticoid expression, which plays an important role in regulating the biosynthesis and metabolism of carbohydrates, lipids, and proteins [14]. Additionally, MnSOD is the primary antioxidant that scavenges superoxide formed within the mitochondria and protects against oxidative stress [15, 16]. If mitochondria are impaired or dysfunctional, ROS production will be further increased and will exacerbate the oxidative stress in mitochondria [17] (Figure 2).

Nevertheless, research in molecular biology or population related to the role of Mn in procession of metabolic diseases via mitochondrial oxidative stress is limited and inconsistent. Mn deficiency and intoxication are both associated with adverse metabolic and neuropsychiatric effects [18, 19]. Experimentally induced Mn deficiency caused a number of detrimental effects, such as impaired growth, poor bone formation and skeletal defects, reduced fertility and birth defects, abnormal glucose tolerance, and altered lipid and carbohydrate metabolism in both animals and humans [2]. By inhibiting mitochondrial complex I and II respiration as well as inducing permeability transition, excessive Mn accumulated in mitochondria could disrupt mitochondrial homeostasis and cause mitochondrial dysfunction [20–22]. In the study about metabolic gene polymorphisms and susceptibility to occupational chronic manganism, it has been found that individuals with homozygote polymorphism (L/L) of the cytochrome P450 2D6L gene (*CYP2D6L*) might decrease the risk of chronic manganism compared with the wild type (Wt/Wt) [23].

In this review, we summarize current hypotheses and research to explore the relationship between Mn and metabolic diseases and reveal how Mn affects the metabolism in both molecular biology and population studies.

## 2. Mn and Metabolic Syndrome

The prevalence of MetS is increasing throughout the world [24]. Recently, criteria to define MetS have been steeped in controversy, but MetS is generally defined by five components: central obesity, raised triglycerides, reduced HDL-cholesterol, raised blood pressure, and raised fasting plasma glucose [25]. The most important role of MetS is to help identify high-risk individuals of both T2DM and cardiovascular disease (CVD) [25].

Oxidative stress is a common risk factor for the pathogenesis of MetS components. Insulin resistance is generally accepted as the first level of metabolic changes in patients with MetS, while the state of chronic low-level inflammation and oxidative stress are second-level abnormalities [26]. Oxidative stress has been associated with all the individual components of MetS and with the onset of cardiovascular complications in subjects with MetS [26–29].

So far, a few researches explore the association between Mn and MetS (Table 1). Higher Mn intake was associated with decreased risk of MetS in men but increased risk in women; Chinese researchers also found that Mn intake was inversely associated with MetS components including abdominal obesity and hypertriacylglycerolaemia in men, but positively associated with low HDL-cholesterol in both men and women [30]. In Korean women with MetS, dietary Mn intake was significantly lower than that of the healthy control group; the same result was also found in women subjects with high blood pressure only [31]. Moreover, another Chinese study indicated that daily intake of Mn was lower in individuals with a higher number of MetS components and a lower risk of developing MetS in the second, third, and highest quintiles of Mn intake compared to the lowest quintile, adjusted for age, sex, and energy intake [32]. However, blood and urine Mn concentrations were not significantly associated with MetS [5, 33].

However, these epidemiologic studies did not consider potential confounding factors, such as changing dietary habits of patients based on their nutritional knowledge about the MetS components, and did not exclude the MetS patients who have accepted therapy. That might be the main causes of previous data showing an inverted relation between Mn intake and risk for MetS. Besides, it is difficult to confirm the association between dietary intake and MetS risk, because the bioavailability of dietary nutrients would be influenced by some factors, for instance, characteristics of the food source, interactions with other dietary factors, cooking conditions, and various subject characteristics.

## 3. Mn and Type 2 Diabetes Mellitus/Insulin Resistance

T2DM accounts for over 90% of global diabetes cases compared to type 1 diabetes. T2DM is characterized by hyperglycemia caused by insulin resistance and/or abnormal insulin secretion, either of which may predominate [34].

Several pathogenic pathways activated in diabetes such as ROS, which are generated by high glucose levels, are responsible for metabolic abnormalities and chronic complications [35]. Moreover, oxidative stress can result in impaired islet beta cell function, cause insulin resistance, and finally lead to T2DM and obesity [7, 8]. Normalizing levels of mitochondrial ROS prevents three pathways of hyperglycaemic damage including glucose-induced activation of protein kinase C, formation of advanced glycation end-products, sorbitol accumulation, and NF*κ*B activation [36]. Mitochondrial dysfunction has divergent, cell type-dependent effects on insulin action [37] and has been proposed to induce insulin resistance through ectopic lipid accumulation secondary to reduced *β*-oxidation, which impairs insulin signaling [38, 39]. In heterozygous MnSOD knockout mice, the MnSOD protein decreased by approximately 70% in muscle and fat, and glucose tolerance was already impaired after feeding these mice a standard chow [40]. Recent studies using transgenic mice that overexpress MnSOD showed protection against diabetic complications, for example, diabetic cardiomyopathy [41], retinopathy [42, 43], and neuropathy [44], while also improving the viability of islet cell transplantation [45]. Therefore, it is very important to maintain the normal function of mitochondrial oxidative stress to prevent the development of T2DM and insulin resistance.

In a study on Zucker rats, a higher mean plasma Mn level in the diabetic fatty group was related to enhanced oxidative stress in diabetes and obesity [46]. Researchers have shown that Mn treatment can increase insulin secretion to improve glucose tolerance under conditions of dietary stress [47], reduce oxidative stress (ROS) and NADPH oxidase [48], and lower the risk of endothelial dysfunction in diabetes [48, 49]. A study on nonobese diabetic mice also found that Mn porphyrin catalytic antioxidant (MnP) treatment slightly enhanced glucose oxidation and reduced fatty acid oxidation [50]. Not only dietary Mn deficiency but also acute Mn exposure in rats can cause decreased plasma insulin levels, rapid hyperglycemia, and hypoinsulinemia, followed by a reactionary hypoglycemia, supporting evidences that the effects of Mn on carbohydrate metabolism may be due to a direct effect on insulin release and gluconeogenesis [51, 52]. A biochemical assessment in male rats' plasma samples showed that MnO_2_ micro- and nanoparticles after injection of subchronic doses significantly increased plasma glucose and cholesterol levels [53].

Several epidemiologic studies have reported direct associations between Mn level and T2DM, although it remains unclear whether Mn plays a positive or negative role (Table 2). Current research suggests that the blood Mn level is significantly increased in T2DM patients [54, 55], while some showed decreased levels [3, 56–59] or even no difference in Mn levels compared to the controls [60]. A case-control study of 3228 participants in China indicated a U-shaped association between plasma Mn and T2DM, with both low and high levels of plasma Mn associated with higher odds of newly diagnosed T2DM [61]. Some research has found a positive correlation between urinary Mn level and T2DM [56, 59]. However, urinary Mn levels of coke oven workers were associated with hyperglycemia risk but not with diabetes risk, which might be due to the small sample size of diabetes and the relatively young population; researchers also found that the concentrations of urinary Mn in the occupational population were higher than those in the general population [62, 63]. Moreover, results were inconsistent in some studies concerning the Mn concentration in the samples of scalp hair, tears, and lymphocytes among individuals with T2DM [56, 59, 64–66].

## 4. Mn and Obesity

Over the last several decades, obesity, defined as excessive fat accumulation, has become an increasingly prevalent metabolic disease [67, 68] that is associated with increased risk of developing T2DM, cardiovascular disease, and NAFLD. Oxidative stress and production of ROS have been linked to the development of insulin resistance, T2DM, and obesity [7, 8, 69], suggesting a potential role for ROS in the pathogenesis of these disorders. In mouse 3T3-L1 mature adipocytes, there is an increased generation of superoxide and higher expression of antioxidant enzymes, potentially to help balance cellular ROS [70, 71]. In the presence of high ROS production, the antioxidant capacity of adipose tissue is also impaired in mouse models of obesity, and antioxidants such as SOD mimetics exert beneficial effects in metabolic diseases associated with obesity [72–75].

Compared with those fed a normal diet, rats fed a high fat-cholesterol diet had a significant decrease in MnSOD activity [76]. It has been shown that MnSOD deletion in mouse adipocytes triggers an adaptive stress response that activates mitochondrial biogenesis and enhanced mitochondrial fatty acid oxidation, thereby preventing diet-induced obesity and insulin resistance [77]. On the other hand, inflammation and excess triglyceride storage induced in obesity mice would raise epididymal adipocyte MnSOD [78]. In mouse studies, manganese [III] tetrakis [5,10,15,20]-benzoic acid porphyrin (MnTBAP), a nonpeptidic mimic of MnSOD, significantly reduced excess body weight and serum superoxide anion generation [79], ameliorated preexisting obesity, and improved insulin action by reducing caloric intake [80]. However, regarding the effect of MnTBAP on adiposity mice and in vivo insulin action, the evidences were conflicting. One suggested a preventive effect on the development of systemic insulin resistance and diabetes after high-fat diet, while the other was not [75, 81].

The concentrations of Mn in the liver, small intestine, and bone of obese mice were significantly lower than those in lean mice [82]. The cross-sectional epidemiological survey has found that plasma Mn was directly correlated with the consumption of dairy products [83]. Higher Mn intake (e.g., >5.12 mg/d) was associated with reduced risk of abdominal obesity and hypertriacylglycerolaemia among men in China [30]. Poland researchers have found that plasma Mn concentration was significantly higher in obese men aged 50–75 years [5]. Nevertheless, the data about blood Mn level in obese children are not consistent [84, 85]. The US National Health and Nutrition Examination Survey 2011–2014, performed with 5404 children and adolescents aged 6–19 years, revealed that the highest blood Mn concentration was associated with obesity and overweight [86].

## 5. Mn and Atherosclerosis

Atherosclerosis is the disease of the arterial wall, characterized by cholesterol accumulation, and culminates in potentially life-threatening conditions, such as heart attack, stroke, and angina [87]. Recent evidence suggests that atherosclerosis is a chronic inflammatory disease of the blood vessel wall [88–90]. Oxidized low-density lipoprotein (oxLDL) and endothelium dysfunction play a key role in the pathogenesis of atherosclerosis [91, 92]. Accumulation of oxLDL in the arterial wall is a characteristic feature of disease progression [88].

Previous studies have demonstrated that the roles of oxLDL and endothelium dysfunction are closely related to the imbalance of oxidative stress and inflammation in the pathogenic process of atherosclerosis [9, 10, 93, 94]. Mitochondrial DNA damage may result from reactive species production in vascular tissues and may in turn be an early event in the initiation of atherosclerotic lesions [95]. MnSOD was reported to reduce the oxLDL-induced apoptosis of macrophages [87, 96], protect against endothelial dysfunction [97, 98], and inhibit the oxidation of LDL by endothelial cells [9]. Furthermore, the association of decreased activity of MnSOD with atherogenesis has suggested that analysis of Mn content in the vascular wall matrix may be one of prospective methods for the diagnosis of early stages of atherosclerosis [99].

Several studies indicated that Mn supplementation could reduce high glucose-induced monocyte adhesion to endothelial cells and endothelial dysfunction and also lower blood levels of ICAM-1 and cholesterol [48, 49], elicit anti-inflammatory effects in endothelial cells [100], and potentially prevent or delay the progression of atherosclerosis. Little is known about the Mn concentration in atherosclerosis patient samples. It has been observed that the difference between the Mn contents of normal and atherosclerotic aortic tissue was not significant [101]. However, in epidemiologic studies, higher blood Mn levels were found in senior citizens aged 61–100 years with atherosclerosis compared to those without [60]. The same result was found in individuals aged 30–62 years [102].

## 6. Mn and Nonalcoholic Fatty Liver Disease

NAFLD, characterized by excess triglyceride (TG) accumulation in the absence of excessive alcohol intake, is the most common chronic liver disease and associated with MetS, obesity, and T2DM [103, 104]. This disease can progress to inflammatory nonalcoholic steatohepatitis (NASH), fibrosis, cirrhosis, and end-stage liver injury in humans [105, 106]. NASH, defined as a necroinflammatory disorder with fatty infiltration of hepatocytes, may progress to fibrosis and lead to cirrhosis [105]. Moreover, nonalcoholic steatosis is the first step in the pathogenesis of NASH linked to mitochondrial dysfunction and oxidative stress [11, 12, 107–109]. Rat histopathological observations suggest that nonpeptidyl mimics of MnSOD may help in the prevention and treatment of NASH in humans [79, 110]. However, few researchers have focused on the association between Mn concentration and NAFLD. In an in vitro NAFLD model established in human SMMC-7721 cells, Mn concentration did not significantly change in oleic acid-induced hepatic steatosis cells compared to the control [111].

## 7. Conclusions

Metabolic diseases are affected by dietary habits, the environment, and genes independently and through their interactions. They are complex diseases caused by multiple etiologies.

Intracellular homeostasis of Mn is associated with some metals. The Mn concentration also affects the absorption and metabolism of other metals. For example, Mn competes for iron (Fe) transporters by inhibiting divalent metal transporter 1 (DMT1) binding with Fe and disrupting the homeostasis of cesium (Cs), cobalt (Co), lead (Pb), mercury (Hg), nickel (Ni), and zinc (Zn) in cells [112]. Researchers have found that Fe depletion increases uptake and potentiates Mn-induced apoptosis, indicated by increased terminal deoxynucleotidyl transferase-mediated dUTP nick end labeling (TUNEL) staining of rat olfactory bulb and human SH-SY5Y cells [113]. Thus, low Fe levels could result in greater absorbance and accumulation of Mn, further influencing its toxicity [114, 115]. Moreover, Mn, copper (Cu), and Zn also competitively combined with SOD in oxidative stress. Alternatively, Mn exposure leads to increased Cu levels and decreased Fe and Ca levels in *Caenorhabditis elegans* (*C. elegans*) [116]. Therefore, studies about mixtures of metals are needed to better clarify how they crosstalk in metabolic diseases.

MnSOD plays a key role in mitochondrial oxidative stress, while the MnSOD Val16Ala polymorphism (rs4880) could result in reduced MnSOD activity and less efficient transport of MnSOD into the mitochondrial matrix [117, 118]. Both the MnSOD gene and levels of Mn could affect the activity of MnSOD [119]. Moreover, Mn supplementation enhanced MnSOD activity and protected against T2DM and its complications [47, 49]. Consequently, it is very important to systematically analyze whether the association with the risk of metabolic diseases and Mn levels is modified by genetic variation in MnSOD, Cu/ZnSOD, and related genes associated with Mn uptake, transport, metabolism, and excretion, such as DMT1, transferrin receptor (TfR), and soluble carrier family (SLC).

Several vitamins are antioxidative compounds, for example, vitamin C, vitamin D3, vitamin E, and *β*-carotene. The human-derived Caco-2 cell study indicated that expression of the SLC30A10 gene, as well as its encoded protein, the Zn and Mn transporter ZnT10, was augmented by vitamin D3 treatment [120]. MnSOD activity was significantly increased with high doses (30 and 100 mg/kg) of vitamin E after 4 and 6 weeks [121]. Thus, it is worth considering whether there is a causal relationship between Mn level and vitamin levels in the process of oxidation.

Previous researches have had small sample sizes, were designed primarily as cross-sectional and case-control studies, and lack large sample prospective studies. Therefore, a cohort study is urgently needed to confirm the causality between Mn and metabolic diseases, especially in occupational Mn-exposed workers [122]. In addition, by using a biological model study, for example, zebrafish and *C. elegans* [123, 124], we can further verify the effects of Mn and the combined action of various metals on metabolic diseases that were found in previous epidemiologic studies.

In summary, Mn is both a toxic and an essential trace element involved in human health and development. In the current literature, research supports a view that a U-shaped association exists between Mn, either deficiency in dietary Mn or excessive Mn exposure, and increased ROS generation as well as oxidative stress, which might affect the occurrence of metabolic diseases further, although it remains inadequate in molecular and epidemiological data on disease patients, especially among Mn workers.

## Figures and Tables

**Table 1:** The studies of Mn and MetS.

Reference	Country	Study design	Sample size	Data source/sample type	Results	
[30]	China	The 5th Chinese National Nutrition and Health Survey (2010–2012)	2111	Questionnaire of dietary Mn intake	Men	A decreased risk of MetS with higher Mn intake.
					Women	An increased risk of MetS with higher Mn intake.
					MetS components	Mn intake was inversely associated with abdominal obesity and hypertriacylglycerolaemia in men, but positively associated with low HDL-cholesterol in both men and women.
[31]	Korea	The Korea National Health and Nutrition Examination Survey (2007–2008)	5136	Questionnaire of dietary Mn intake	Men	No difference
					Women/MetS components	The women subjects with high blood pressure showed significantly lower intake of Mn than did control subjects.
[32]	China	Cross-sectional study	Cases: 221 Controls: 329	Questionnaire of dietary Mn intake	Men/women	A lower risk of developing MetS in the second, third, and highest quintiles of Mn intake with respect to the lowest quintile after adjusting age, sex, and energy intake.
					MetS components	Daily intake of Mn was decreased with the increasing number of MetS components.
[5]	Poland	Cross-sectional study	313 (men aged 50–75 years)	Serum (Mn level)	Significant positive correlations (Mn–BMI, Mn–abdominal circumference, Mn–waist-to-hip ratio, Mn–insulin, Mn–HOMA-IR), but no correlation with MetS.	
[33]	Korea	The Korea National Health and Nutrition Examination Survey (2008)	1405	Whole blood (Mn level)	No difference	
				Urine (Mn level)	No difference	

**Table 2:** The epidemiologic studies of Mn level in T2DM, obesity, and atherosclerosis.

Reference	Country	Disease	Study design	Sample size	Sample type	Results of Mn level in cases
[3]	Korean	T2DM	Cross-sectional study	3996	Whole blood	Decreased
[54]	Mexico	T2DM	Cross-sectional study	76	Serum	Increased
					Urine	No different
[55]	Turkey	T2DM	Hospital-based case-control study	Cases: 200 Controls: 50	Serum	Increased
[56]	Pakistan	T2DM	Cross-sectional study	Diabetes: 257 Healthy: 166	Whole blood	Decreased
					Urine	Increased
					Scalp hair	Decreased
[57]	Egypt	T2DM	Hospital-based case-control study	Cases: 40 Controls: 36	Serum	Decreased
[58]	Italy	T2DM	Case-control study	Cases: 68 Controls: 59	Whole blood	Decreased
[59]	Pakistan	T2DM	Hospital-based case-control study	Cases with their infants: 76 Healthy with their infants: 68	Whole blood	Decreased
					Urine	Increased
					Scalp hair	Decreased
[60]	Czech Republic	T2DM	Cross-sectional study	1069 (aged 61–100 years)	Whole blood	No different
[61]	China	T2DM	Case-control study	Cases: 1614 Controls: 1614	Plasma	U-shaped association
[62]	China	T2DM	Cross-sectional study	1493 (coke oven workers)	Urine	Increased association with hyperglycemia risk but not with diabetes risk
[64]	Pakistan, Ireland	T2DM	Case-control study	Cases: 145 Controls: 177	Scalp hair	Decreased
[65]	Austria	T2DM	Hospital-based case-control study	Cases: 53 Controls: 50	Lymphocyte	Decreased
[66]	Italy	T2DM	Case-control study	Cases: 47 Controls: 50	Tear	Increased
[5]	Poland	Obesity	Cross-sectional study	313 (men aged 50–75 years)	Serum	Increased
[83]	Spain	Obesity	Cross-sectional study	340	Plasma	Increased association with the consumption of dairy products
[30]	China	Obesity	Cross-sectional study	2111	None	Higher Mn intake (e.g., >5.12 mg/d) was associated with a reduced risk of abdominal obesity and hypertriacylglycerolaemia among men.
[84]	Turkey	Obesity	Hospital-based case-control study	Cases: 57 Controls: 48 (children aged 6–17 years)	Serum	Increased
[85]	Turkey	Obesity	Prospective observational study	Cases: 34 Controls: 33 (children)	Serum	No different
[86]	USA	Obesity	Cross-sectional study	5404 (children and adolescents aged 6–19 years)	Serum	Increased
[60]	Czech Republic	Atherosclerosis	Cross-sectional study	1069 (aged 61–100 years)	Whole blood	Increased
[102]	Pakistan	Atherosclerosis	Case-control study	Cases: 90 Controls: 90 (aged 30–62 years)	Blood	Increased
